# Nightmare-Induced Atypical Midventricular Tako-Tsubo Cardiomyopathy

**DOI:** 10.1155/2015/292658

**Published:** 2015-02-19

**Authors:** Veronica Fibbi, Piercarlo Ballo, Marco Nannini, Lorenzo Consoli, Tania Chechi, Andrea Bribani, Francesca Fiorentino, Leandro Chiodi, Alfredo Zuppiroli

**Affiliations:** ^1^Cardiology Unit, S. Maria Annunziata Hospital, 50012 Florence, Italy; ^2^Emergency Department, Serristori Hospital, Figline Valdarno, 50063 Florence, Italy; ^3^Emergency Department, S. Maria Annunziata Hospital, 50012 Florence, Italy

## Abstract

Tako-Tsubo cardiomyopathy (TTC) is a reversible cardiomyopathy characterized by acute left ventricular segmental dysfunction, whose clinical presentation resembles that of acute myocardial infarction. The syndrome often follows a psychophysical stressful event and is characterized by echocardiographic evidence of akinesia of the left ventricular mid-apical segments. Atypical echocardiographic patterns of TTC have recently been described, often triggered by emotional stressors, rather than physical. In this report, we describe a case of atypical TTC triggered by an unusual stressor (recurrent nightmare) in a 45-year-old woman, with peculiar clinical presentation and evolution characterized by persistent loss of consciousness, neurological deterioration, absence of typical symptoms of TTC, and features suggestive of a hysterical crisis.

## 1. Introduction

Tako-Tsubo cardiomyopathy (TTC), also called broken heart syndrome or apical ballooning, is a reversible cardiomyopathy characterized by acute left ventricular (LV) segmental dysfunction, often precipitated by a psychophysical stressful event, whose clinical presentation mimics that of acute myocardial infarction [1]. TTC most commonly occurs in women, particularly in the postmenopausal period, and is estimated to represent about 1-2% of subjects presenting with troponin-positive acute coronary syndrome [2]. The classic pattern of TTC is characterized by akinesia of the mid-apical segments of LV walls, often with hyperkinesia of the basal segments. The resulting shape of LV cavity typically shows a round bottom and narrow neck, resembling that of a traditional Japanese octopus trap called “Tako-Tsubo.” Atypical clinical and echocardiographic patterns of TTC in which the diagnosis may be difficult to make, often leading to misdiagnosis, have recently been described [3]. Although several types of triggering event have been previously described, identification of the stressor may sometimes be challenging, as up to one-third of TTC patients present with no clear evidence of definite stressful event [4]. In this report, we describe a case of TTC with atypical clinical and echocardiographic features, which was triggered by an unusual stressor, that is, a recurrent nightmare.

## 2. Case Report

**Figure 1:**
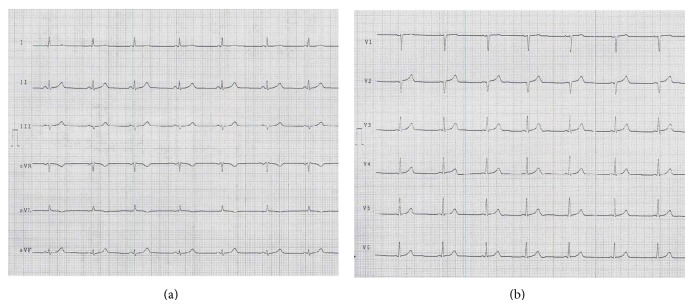
Electrocardiogram at admission showing nonsignificant, ascending ST-elevation in right precordial limbs, with no evidence of mirror images. Also note the negative T wave in aVL.

**Figure 2:**
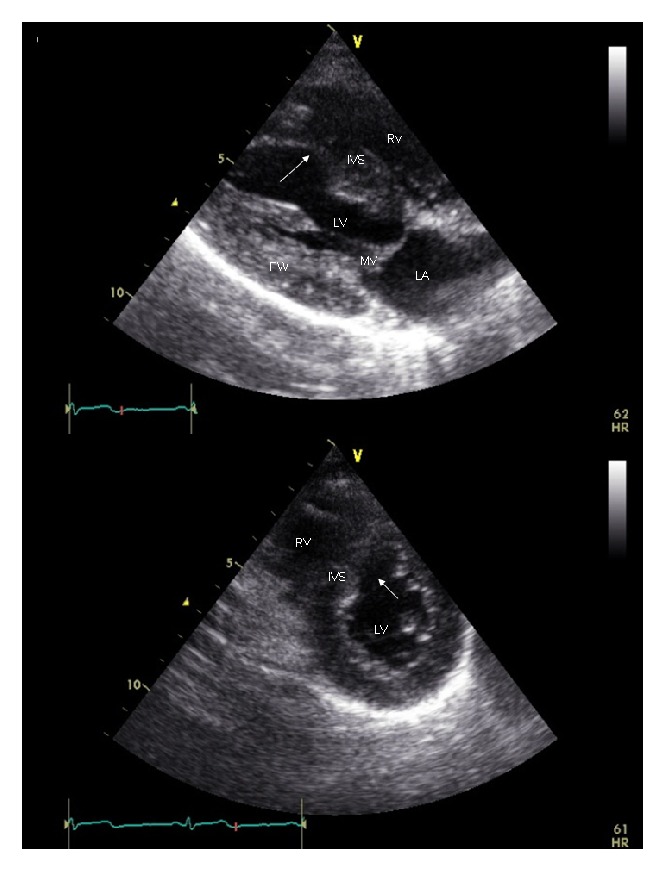
Echocardiographic images taken during ventricular systole from the parasternal long-axis view (top panel) and short-axis view (bottom panel), showing akinesia of the middle segment of the ventricular septum (arrows). Note the lack of systolic thickening of the akinetic segment, as compared to the others. IVS: interventricular septum; LA: left atrium; LV: left ventricle; MV: mitral valve; PW: posterior wall of the left ventricle; RA: right atrium; RV: right ventricle.

A 45-year-old woman was urgently brought to the Emergency Department because of persistent loss of consciousness. Her history was unremarkable, except for anxiety-depressive disorder. She had no cardiovascular risk factors except for current cigarette smoking, and she was not assuming any type of cardiovascular or noncardiovascular medication. Two hours earlier, she had phoned the Medical Emergency Team because of general discomfort and intense anxiety. She had been administered benzodiazepines, but the discomfort had worsened with progressive neurological deterioration. At admission, chest and heart examination was normal, blood pressure was 100/70 mmHg, and heart rate was 70 bpm. On neurological examination, there were spontaneous eye opening, absence of verbal response, and withdrawal from painful stimuli (Glasgow coma scale 9), arm and leg extension, lock jaw, hypersalivation, and diffuse hyporeflexia. The electrocardiogram showed sinus rhythm, a nonsignificant (<1 mm) ST-elevation with ascending slope in right precordial limbs, with no evidence of mirror images, and a negative T wave in aVL (Figures 1(a) and 1(b)). Echocardiography showed normal left ventricular (LV) ejection fraction (55%) with akinesia of the middle segments of ventricular septum and both anterior and inferior walls (Figure 2, Movie 1 in Supplementary Material available online at http://dx.doi.org/10.1155/2015/292658). Blood examinations showed increased levels of troponin I (1.99 ng/mL, n.v. < 0.09 ng/mL) and D-dimer (4069 ng/mL, n.v. < 500 ng/mL) and leukocytosis (11.0 · 10^3^/*μ*L). Arterial blood gas analysis showed mild compensated metabolic acidosis with hyperlactatemia (7.4 mmol/L, n.v. 0.3–0.8 mmol/L). During the stay in the Emergency Department, the patient developed convulsive seizures, which were treated with i.v. diazepam. An electroencephalogram and brain computer tomography showed normal findings. The patient was transferred to the Intensive Care Unit with the diagnosis of acute coronary syndrome. Treatment with aspirin, clopidogrel, fondaparinux, atorvastatin, and pantoprazol was started. Low-dosage metoprolol was started on day 2 but was discontinued the day after because of low blood pressure. Because the patient showed preserved LV ejection fraction, no symptoms or signs of heart failure, and a tendency toward hypotension, we also did not start ACE-inhibitors, angiotensin receptor blockers, or diuretics.

**Figure 3:**
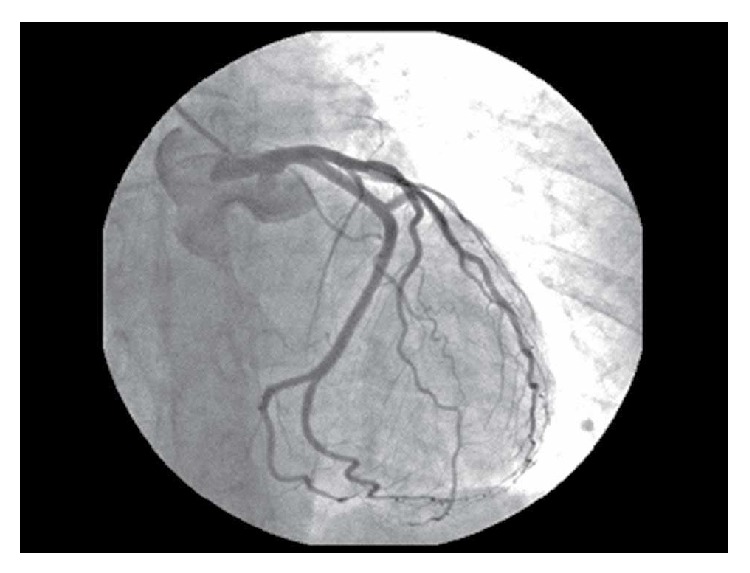
Angiographic image showing normal coronary arteries.

Few hours later, progressive recovery of consciousness was observed, with retrograde amnesia and a transient phase of behaviour abnormalities with histrionic issues. The day after, following full recovery of consciousness, the patient referred that during the previous five days she had experienced a recurrent nightmare with sudden awakenings characterized by severe anxiety, prolonged tachycardia, and sweating. Although she remembered the frightening experience associated with the nightmare, she was not able to remember its contents. An angiography showed normal coronary arteries (Figure 3). The electrocardiogram showed development of negative T waves in V1-V2. Blood examinations showed increased NT-proBNP plasma concentration (2514 pg/mL, n.v. < 125 pg/mL), a borderline level of C-reactive protein (0.52 mg/dL, n.v. < 0.50 mg/dL), and progressive normalization of troponin I. Patient's general conditions rapidly improved. Atypical midventricular TTC was diagnosed. She was discharged on aspirin 100 mg od, with the advice of continuing it indefinitely. On day 10, echocardiography showed improvement of LV systolic function with residual hypokinesia of the middle segments of the septum and anterior and inferior wall. One month later, segmental wall motion was completely restored.

## 3. Discussion

According to the modified Mayo Clinic criteria, the diagnosis of TTC is based on the following findings: (1) transient hypokinesis, dyskinesis, or akinesis of the LV midsegments, with or without apical involvement, and with regional wall-motion abnormalities extending beyond a single epicardial vascular distribution; (2) absence of obstructive coronary disease or angiographic evidence of acute plaque rupture; (3) new ECG abnormalities (ST-segment elevation and/or T wave inversion) or modest elevation in cardiac troponin; and (4) absence of pheochromocytoma and myocarditis [5]. Although most common presenting symptoms include chest pain and dyspnea, several other presentations have been reported, such as syncope, asthenia, arrhythmias including ventricular tachycardia and ventricular fibrillation, and sudden death [6–9]. Potential complications of TTC include heart failure [10], bradyarrhythmias [11], cardiogenic shock [12], LV outflow tract obstruction induced by LV basal hyperkinesis [13], and acute mitral regurgitation [14]. The possibility that TTC could favour LV apical thrombus formation due to transient LV wall motion abnormalities, leading to an indication to anticoagulant therapy, has also been reported [15]. The exact etiology of TTC is still unknown. Possible pathophysiological mechanisms include multivessel epicardial coronary artery spasm, abnormalities in microvascular function, endogenous catecholamine-induced myocardial stunning and/or toxicity, impaired myocardial fatty acid metabolism, and acute coronary syndrome with reperfusion injury [16]. The classic echocardiographic pattern of TTC is characterized by mid-apical ballooning, but atypical variants such as reverse TTC (with involvement of LV basal segments) or midventricular TTC (with involvement of LV midventricular segments and preservation of apical wall motion) have been described [17]. Atypical variants of TTC have been reported to account for about 40% of cases [18].

A significant emotional or physical stressor typically precedes the development of TTC. A high number of stressors able to induce TTC have been reported, including death of a loved one, legal disputes, bad financial news, natural disasters such as earthquakes, tempests, and floods, car accidents, strenuous physical efforts, exacerbation of a chronic medical illness, newly diagnosed diseases, surgical interventions, hospital stay in an intensive care unit, seizure, migraine, asthmatic crisis, carbon monoxide poisoning, use of or withdrawal from illicit drugs, and suicide attempt [19–22].

To our knowledge, this is the first report of midventricular TTC triggered by a recurrent nightmare. Several atypical issues should be pointed out in this report: (1) the unusual type of stressor; (2) the clinical presentation, characterized by persistent loss of consciousness, neurological deterioration despite normality of electroencephalogram and brain computer tomography, and absence of typical symptoms of TTC; and (3) the early clinical evolution, which might suggest the typical pattern of an hysterical crisis, characterized by an aura followed by epileptoid, histrionic, and recovery phases. Although the loss of consciousness followed by retrograde amnesia cannot allow excluding the fact that our patient also experienced chest pain or dyspnea, it is interesting to observe that TTC triggered by emotional stressors, rather than physical, often tends to present without typical symptoms [23]. Another interesting issue is that seizures have been reported as causes of TTC [24]. In particular, it has been reported that patients with seizure-associated TTC tend to be younger, more frequently males, and often present with no chest pain but frequent cardiogenic shock and sudden hemodynamic deterioration [25]. Although most of these characteristics were not present in our patient, it cannot be excluded that the hysterical episode that occurred in our patient, and particularly the convulsive phase, may have played a role as an additional trigger.

## Supplementary Material

Supplementary Movie: Short-axis echocardiographic movie showing akinesia of the middle septum.
